# Transcranial Magnetic Stimulation of the Default Mode Network to Improve Sleep in Individuals With Insomnia Symptoms: Protocol for a Double-Blind Randomized Controlled Trial

**DOI:** 10.2196/51212

**Published:** 2024-01-26

**Authors:** Lindsey Hildebrand, Alisa Huskey, Natalie Dailey, Samantha Jankowski, Kymberly Henderson-Arredondo, Christopher Trapani, Salma Imran Patel, Allison Yu-Chin Chen, Ying-Hui Chou, William D S Killgore

**Affiliations:** 1 Department of Psychiatry University of Arizona Tucson, AZ United States; 2 Department of Psychology University of Arizona Tucson, AZ United States; 3 Division of Pulmonary, Allergy, Critical Care and Sleep Medicine University of Arizona Tucson, AZ United States

**Keywords:** continuous theta burst stimulation, transcranial magnetic stimulation, default mode network, sleep, insomnia, cTBS, randomized controlled trial

## Abstract

**Background:**

Cortical hyperarousal and ruminative thinking are common aspects of insomnia that have been linked with greater connectivity in the default mode network (DMN). Therefore, disrupting network activity within the DMN may reduce cortical and cognitive hyperarousal and facilitate better sleep.

**Objective:**

This trial aims to establish a novel, noninvasive method for treating insomnia through disruption of the DMN with repetitive transcranial magnetic stimulation, specifically with continuous theta burst stimulation (cTBS). This double-blind, pilot randomized controlled trial will assess the efficacy of repetitive transcranial magnetic stimulation as a novel, nonpharmacological approach to improve sleep through disruption of the DMN prior to sleep onset for individuals with insomnia. Primary outcome measures will include assessing changes in DMN functional connectivity before and after stimulation.

**Methods:**

A total of 20 participants between the ages of 18 to 50 years with reported sleep disturbances will be recruited as a part of the study. Participants will then conduct an in-person screening and follow-on enrollment visit. Eligible participants then conduct at-home actigraphic collection until their first in-residence overnight study visit. In a double-blind, counterbalanced, crossover study design, participants will receive a 40-second stimulation to the left inferior parietal lobule of the DMN during 2 separate overnight in-residence visits. Participants are randomized to the order in which they receive the active stimulation and sham stimulation. Study participants will undergo a prestimulation functional magnetic resonance imaging scan and a poststimulation functional magnetic resonance imaging scan prior to sleep for each overnight study visit. Sleep outcomes will be measured using clinical polysomnography. After their first in-residence study visit, participants conduct another at-home actigraphic collection before returning for their second in-residence overnight study visit.

**Results:**

Our study was funded in September 2020 by the Department of Defense (W81XWH2010173). We completed the enrollment of our target study population in the October 2022 and are currently working on neuroimaging processing and analysis. We aim to publish the results of our study by 2024. Primary neuroimaging outcome measures will be tested using independent components analysis, seed-to-voxel analyses, and region of interest to region of interest analyses. A repeated measures analysis of covariance (ANCOVA) will be used to assess the effects of active and sham stimulation on sleep variables. Additionally, we will correlate changes in functional connectivity to polysomnography-graded sleep.

**Conclusions:**

The presently proposed cTBS protocol is aimed at establishing the initial research outcomes of the effects of a single burst of cTBS on disrupting the network connectivity of the DMN to improve sleep. If effective, future work could determine the most effective stimulation sites and administration schedules to optimize this potential intervention for sleep problems.

**Trial Registration:**

ClinicalTrials.gov NCT04953559; https://clinicaltrials.gov/ct2/show/NCT04953559

**International Registered Report Identifier (IRRID):**

DERR1-10.2196/51212

## Introduction

### Overview

The sleep disturbances of insomnia include difficulty falling asleep, staying asleep, or inability to fall back to sleep after premature awakenings, or any combination of these, along with daytime sleepiness and dysfunction. Difficulty falling and remaining asleep affected nearly 30% of the US population, with approximately 20% of the general population experiencing occasional difficulties and an additional 10% meeting the clinical criteria for an insomnia disorder [1]. However, this protocol was conducted during the COVID pandemic, when the prevalence of insomnia symptoms increased to 53% and insomnia disorder prevalence jumped from 10% to 17% [2]. Insomnia symptoms have been linked with poorer health across multiple domains and exacts a high societal cost in the workplace, on the health care system, and on an individual’s and family’s quality of life [3-5]. Moreover, side effects from medications used to manage insomnia increase these factors in some cases. Novel approaches that facilitate sleep are critically needed, and some potential neural mechanisms of insomnia create a viable treatment target [6].

The most widely accepted theory of primary insomnia is the hyperarousal hypothesis, which suggests that problems with sleep initiation and maintenance are due largely to the disruptive effects of somatic or cognitive hyperarousal [7,8]. In particular, cortical hyperarousal can emerge from excessive focus on repetitive negative thoughts, including intensive problem-solving, self-reflective rumination, and worry [9-11]. Subjectively, people who struggle with insomnia often make comments such as “I wish I could just turn my mind off” or “I just keep replaying conversations over in my head.” In fact, one of the major features of insomnia is the tendency toward self-reflective rumination and worry [9,12-15]. This internal dialogue contributes to a cycle of self-referential thought and hyperarousal that appears to hinder sleep onset and maintenance [9,16-18]. In fact, worry and sleep disruptions are associated in the general population [3]. In individuals with insomnia, cortical hyperarousal contributes to difficulty initiating and maintaining sleep [19].

Neuroimaging research has shown that internally focused self-reflective processes of this type tend to activate an interconnected system within the brain known as the default mode network (DMN) [20,21]. Moreover, negative ruminative thinking is associated with changes in DMN connectivity and other brain regions associated with cognitive arousal or negative emotion [22]. Patients with insomnia often show abnormalities in the functioning of the DMN that are consistent with the hyperarousal hypothesis [23-25]. The core nodes of the DMN typically include the medial prefrontal cortex, posterior cingulate cortex, precuneus, and bilateral inferior parietal cortex regions but can also include several ancillary smaller regions, including the hippocampus, medial temporal lobes, and other subcortical structures [26]. Individuals with insomnia disorder tend to have increased resting-state functional connectivity (FC) between spatially segregated nodes of the DMN [27,28]. Increased connectivity and activation of the DMN could contribute to the ongoing self-referential processing and internal dialogue that maintains a hyperaroused state and perpetuates difficulties falling and remaining asleep [27,29]. Individuals with insomnia disorder show greater activation of the DMN compared with healthy controls while viewing word lists associated with past, present, and future worries, particularly when the words are self-referential [29].

Mainstream approaches to treating primary insomnia include cognitive behavioral therapy for insomnia and pharmacologic sleep aids, such as hypnotic sedatives [30-33]. While cognitive behavioral therapy for insomnia often helps to reduce ruminative cognitions and is effective at improving sleep for many individuals, there are many who fail to achieve meaningful benefits [34,35]. Similarly, pharmacologic sleep aids also tend to have modest effect sizes [36] and are often associated with unwanted side effects (eg, daytime drowsiness and memory problems) and health-related morbidities [37]. Various noninvasive neuromodulatory approaches have been shown to improve insomnia symptoms, including repetitive transcranial magnetic stimulation (rTMS) [38]. However, low-frequency rTMS has a cortical inhibition effect that has been shown to improve insomnia symptoms, which may be due to hyperarousal across multiple psychological and physiological domains [39].

The proposed protocol aims to bridge the gap between cognitive and neuromodulatory interventions to facilitate sleep onset and longer maintenance by temporarily inhibiting the brain FC that is associated with cortical hyperarousal and presleep ruminative cognitions. Our proposed approach will involve using rTMS prior to bedtime to briefly disrupt the strength of FC among cortical regions of the DMN. As we propose to inhibit connectivity within the DMN, we will use continuous theta burst stimulation (cTBS), which induces long-term depression of cortical neural firing following a sustained stimulation period of 40 seconds [40]. Thus, we hypothesize that the application of cTBS to an easily accessible node of the DMN will suppress the local neural activity of that node and propagate inhibition throughout the DMN, thereby reducing ruminative thinking and worry prior to sleep onset. Decreased DMN connectivity is expected to improve sleep quality and quantity. An increase in slow-wave and rapid eye movement sleep stages has been proposed as mechanisms of rTMS in increasing restorative sleep in individuals with insomnia, so polysomnography (PSG) parameters are the primary outcome measures in the outlined protocol [41]. This phase 1 clinical trial will be the first study to investigate the effects of cTBS targeted to the left inferior parietal node of the DMN on objectively measured sleep outcomes.

### Research Aims and Hypotheses

This study aims to explore the effects of cTBS on (1) the activation and connectivity of the DMN and (2) sleep outcomes. We hypothesize that modulating the DMN by stimulating a targeted region of the left inferior parietal lobule with a single cTBS administration will decrease FC within the DMN and thereby improve sleep parameters (ie, PSG) relative to an identical sham administration.

### Study Design and Randomization

This phase 1 clinical trial (ClinicalTrials.gov NCT04953559) will be conducted with a randomized, double-blind, sham-controlled, counterbalanced, crossover design. Participants serve as their own controls as they undergo 2 identical study sessions separated by at least 5 days (ie, washout period). Participants and study personnel who interact with the participants will be blind to the specific condition (active cTBS vs sham) the participant receives during each study visit. Using a prespecified, equally balanced, block randomization procedure, participants will be assigned to the order in which they receive active cTBS and sham stimulation conditions. The randomization list will be generated by a computer random number generator, with the constraint that half of the participants of each biological sex will receive sham first and half will receive the active cTBS first. This design will allow us to examine the intraindividual effects of the active cTBS and establish the superiority of active cTBS compared with sham stimulation in improving PSG sleep outcomes.

## Methods

### Study Procedures Overview

**Figure 1:**
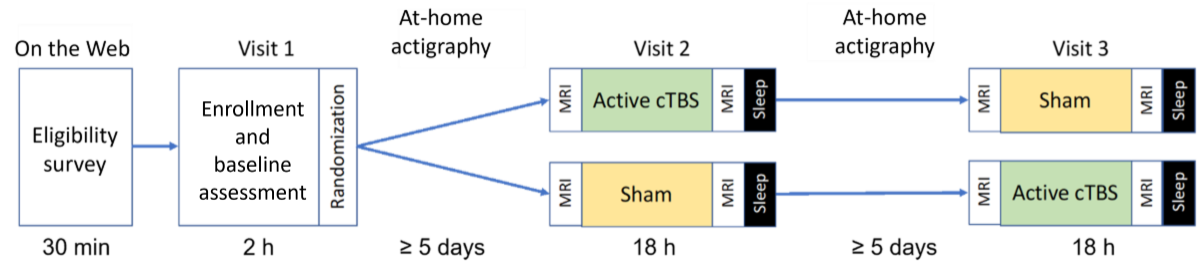
Overview of the pilot randomized controlled trial study design. Participants first complete a web-based eligibility survey screening for sleep disturbances. Potentially eligible volunteers complete an in-lab enrollment process and baseline assessment (visit 1) at a large Southwestern University medical research center. Participants are then randomized to a stimulation order condition. After at least 5 days with actigraphically measured sleep, participants return for an 18-hour overnight session involving 2 MRI scans and overnight sleep (visit 2) at the medical research center. Depending on their condition assignment order, they either receive active cTBS or sham intervention. Participants then undergo a washout period of at least 5 days that includes actigraphically measured sleep. They then return for an identical overnight visit that involves the alternate intervention condition. cTBS: continuous theta burst stimulation; MRI: magnetic resonance imaging.

The study procedures were completed over 3 separate visits. Screening to determine eligibility and baseline assessments was conducted during visit 1. Eligible participants were asked to wear a wrist actigraph for at least 5 days prior to their first in-laboratory overnight visit (visit 2). Based on a preestablished counterbalanced crossover randomization schedule, participants were then assigned to receive either the active cTBS or sham stimulation for their first overnight visit (visit 2) followed by the alternative intervention during their second overnight visit (visit 3) at least 5 days later. As shown in Figure 1, each in-laboratory session lasted 18 hours, including a 2-hour cognitive testing block, 1 hour of preintervention magnetic resonance imaging (MRI), followed by either active cTBS or sham stimulation, and a postintervention hour of neuroimaging. Participants were then fitted with PSG electrodes and were permitted 8 hours of sleep while undergoing PSG sleep monitoring in the lab. The next morning, participants completed another 2-hour cognitive testing block before being released.

The full study execution takes approximately 38-40 hours with 2-3 hours allocated for baseline screening and enrollment (visit 1) and 18 hours allocated for each overnight visit (visits 2 and 3). Table 1 presents the specific assessment measures administered at each visit.

**Table 1:** List of study activities and assessments performed during study visits during a pilot randomized controlled trial evaluating the preliminary effectiveness of continuous theta burst stimulation in improving sleep in individuals with reported sleep disturbances.

Study activities and assessments			Baseline screening and enrollment (visit 1)		At home		Overnight visits (visits 2 and 3)
**Screening**							
	Consent form	✓					
	Eligibility screening (TMS^a^, health background, MRI^b^, PSQI^c^, ISI^d^, and ESS^e^)	✓					
	Pregnancy test	✓					
**Covariates**							
	Demographics	✓					
	Anxiety (STAI-S^f^ and STAI-T^g^)	✓				✓	
	Depression (BDI-II^h^)	✓				✓	
	Sleep preoccupation (SPS^i^ and GCTI^j^)	✓				✓^k^	
	Caffeine consumption questionnaire	✓					
	Intelligence (WASI-II^l^)	✓					
	Actigraphy (Phillips Actiwatch)			✓			
	Sleep quality (Sleep diaries)			✓			
**Primary Outcomes**							
	Functional connectivity in DMN^m^					✓	
	Sleep (PSG^n^)					✓	
**Secondary Outcomes**							
	Information processing speed (GNG^o^)					✓	
	Attention (PVT^p^)					✓	
	Verbal memory (CVLT-3^q^)					✓	
	Immediate and delayed memory (RBANS^r^)					✓	
	Self-reported sleepiness (KSS^s^)					✓	
	Mood (VAMS^t^)					✓	
	Side effects (pre- and post-TMS assessment)					✓	

^a^TMS: transcranial magnetic stimulation.

^b^MRI: magnetic resonance imaging.

^c^PSQI: Pittsburgh Sleep Quality Index.

^d^ISI: Insomnia Severity Index.

^e^ESS: Eppworth Sleepiness Scale.

^f^STAI-S: State-Trait Anxiety Inventory-State.

^g^STAI-T: State-Trait Anxiety Inventory-Trait.

^h^BDI-II: Beck Depression Inventory.

^i^SPS: Sleep Preoccupation Scale.

^j^GCTI: Glasgow Content of Thoughts Inventory.

^k^Only GCTI.

^l^WASI-II: Wechsler Abbreviated Scale of Intelligence.

^m^DMN: default mode network.

^n^PSG: polysomnography.

^o^GNG: go/no-go task.

^p^PVT: psychomotor vigilance task.

^q^CVLT-3: California Verbal Learning Task 3.

^r^RBANS: repeatable battery for the assessment of neuropsychological status.

^s^KSS: Karolinska Sleepiness Scale.

^t^VAMS: Visual Analogue Mood Scale.

### Ethical Considerations

All study activities and study personnel were approved by the University of Arizona’s Institutional Review Board (IRB) and by the Department of Defense Office of Human Research Oversight (OHRO) in March 2021 (approval 2007900971). Any protocol amendments and reportable new information were disseminated to both IRB and OHRO. All participant information was deidentified for confidentiality. Participants were compensated 500 dollars via check for their participation in the study if they were fully compliant with the study procedures. The study team maintains dissemination control of the final deidentified data set. Final results will only report cumulative population data to respective regulatory and scientific reporting agencies and along with peer-reviewed publications. Access to the deidentified data set will be available upon request to the principal investigator and in conjunction with proper regulatory requirement.

### Participants and Recruitment

A total of 20 otherwise healthy adults with self-reported sleep disturbances were recruited from the greater Tucson and Phoenix metropolitan areas. Recruitment strategies include flyers posted on community boards, advertisements in local newspapers, sponsored social media posts, and email lists at local universities. Individuals who were recruited from the community were directed to complete a web-based survey in a HIPAA (Health Insurance Portability and Accountability Act)–compliant server. If participants met the initial inclusion criteria, a study member then completed a preliminary phone screening with the participant. Study personnel then scheduled an in-person screening visit for eligible individuals.

### Power Analyses

During the study design, we conducted a power analysis to determine the sample size necessary to compare mean changes in DMN connectivity before and after cTBS using a within-subjects ANOVA approach. From prior cited work on transcranial magnetic stimulation (TMS) and FC, a total sample of 20 was found to have sufficient power to answer the research questions to include analysis of 2 groups (within-subject active and sham), 2 repeated measurements (before and after TMS), and a correlation between the change in DMN activation and sleep outcome metrics [42,43].

### Inclusion and Exclusion Criteria

Individuals between the ages of 18-50 years with reported sleep disturbances were included in the study. Individuals were excluded if they reported or exhibited any health conditions beyond insomnia-related symptoms. Exclusionary criteria were based on one or more of the following considerations: the criterion in question is (1) known to alter sleep, (2) known to substantially increase interparticipant variability, (3) known to put the volunteer outside the range of what is considered healthy, or (4) required by regulation. Detailed inclusion and exclusion criteria are referenced in Textbox 1.

**A detailed description of inclusion and exclusion criteria of a pilot randomized controlled trial evaluating the preliminary effectiveness of continuous theta burst stimulation in improving sleep in individuals with reported sleep disturbances.**

**Inclusion criteria**

- 18-50 years old
- Self-reported sleep disturbances on 2 of 3 inventories:
  Pittsburgh Sleep Quality Index ≥6
  Insomnia Severity Index ≥15
  Epworth Sleepiness Scale ≥11

**Exclusion criteria**

- Unwillingness to provide informed consent
- Presence of a metal implant or medical device which poses a safety risk for magnetic resonance imaging or transcranial magnetic stimulation
- Self-reported past or present medical diagnosis of sleep- or breathing-related disorders
- Travel outside the time zone within 1 week prior to the enrollment visit or while active during the study
- Self-reported major medical or neurological problems
- Self-reported past or present history of cardiovascular disease
- Self-reported past or present history or first-degree family history of any seizures or seizure disorders
- Self-reported underlying acute or chronic pulmonary disease
- Self-reported history of fainting spells or syncope
- Self-reported past or present psychiatric problems
- Self-reported suicidal ideation
- Self-reported current use of prescription medications
- Self-reported current use of supplements that affect sleep
- Self-reported caffeine use in excess of 300 mg per day on average
- Self-reported regular nicotine use
- Self-reported or suspected heavy alcohol indicated on the Alcohol Use Disorders Identification Test (AUDIT) as greater than or equivalent to 14 drinks a week for male individuals and 7 drinks a week for female individuals
- Self-reported use of illicit drugs
- Speaking English as a nonprimary language
- Less than a ninth-grade education
- Engaged in overnight shift work
- Female individuals only: positive urine pregnancy test
- Female individuals only: self-reported current breast-feeding or collecting breast milk

### Study Activities

#### Enrollment and Baseline Assessment (Visit 1)

Eligible participants were invited for an in-person screening and baseline data collection session lasting approximately 2-3 hours. After participant consent was obtained by trained study staff members, the participants underwent a baseline assessment, including measurement of intellectual capacity with the Wechsler Abbreviated Scale of Intelligence by a certified administrator, subjective sleep assessment including the Pittsburgh Sleep Quality Index and Insomnia Severity Index, and other cognitive assessments.

#### At-Home Actigraphy Week

Upon the completion of the enrollment visit, participants were fitted with a wrist actigraphic sleep monitor that they wear for at least 5 days prior to returning for the in-laboratory assessment sessions and continued to wear the device during the intervening washout week between the 2 in-residence laboratory stays. Participants also completed a web-based sleep diary each morning. Throughout the entire period of enrollment, participants were required to maintain a regular sleep schedule and were not permitted to use caffeine products for 48 hours prior to the in-laboratory overnight stays.

#### Overnight In-Residence Laboratory Sessions (Visits 2 and 3)

**Figure 2:**
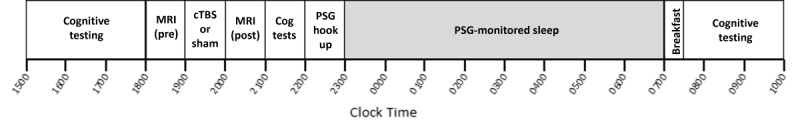
Timeline of the in-residence laboratory testing for the pilot randomized controlled trial. Participants arrive at the lab at 1500 and undergo cognitive testing followed by a preintervention MRI scan. The intervention is administered between 1900 and 2000 and is randomly assigned as either active cTBS stimulation or an identical appearing sham condition. After stimulation, participants complete a postintervention series of MRI scans, followed by a brief cognitive testing battery. At 2200, they are escorted to the sleep lab and PSG electrodes are applied. Lights out occur at 2300 and the participant is provided with an 8-hour undisturbed period for PSG-monitored sleep. Following wake-up at 0700 the next morning, the participant is provided a light breakfast and completes a final battery of cognitive tests. This procedure is repeated on separate weeks for the active cTBS and placebo conditions. cTBS: continuous theta burst stimulation; MRI: magnetic resonance imaging; PSG: polysomnography.

Participants completed 2 overnight in-residence laboratory stays according to a double-blind counterbalanced crossover design, with each session separated by at least 5 days. Figure 2 provides a graphical overview of the in-residence laboratory activities.

#### Cognitive Testing Block

Participants arrived at 3 PM and completed approximately 3 hours of cognitive testing with a trained and certified administrator, including the 10-minute psychomotor vigilance testing (PVT) and Karolinska Sleepiness Scale (KSS) at 3 time points separated by an hour, in addition to the Visual Analog Mood Scale, State-Trait Anxiety Inventory-State only (STAI-S), Beck Depression Inventory (BDI-II), California Verbal Learning Task 3 (CVLT3), go/no-go task (GNG), repeatable battery for the assessment of neuropsychological status (RBANS) symbol digit test, RBANS digit span, RBANS story memory test, and Glasgow Content of Thoughts Inventory (GCTI).

#### Prestimulation Neuroimaging

MRI scans were collected on a Siemens MAGNETOM Skyra 3T scanner (Siemens) using a 32-channel head coil. Participants completed a series of scans that included structural (magnetization-prepared rapid gradient-echo), functional (10-min eyes open) resting state, and proton magnetic resonance spectroscopy (^1^H MRS) scans, with voxels placed in (1) the anterior cingulate gyrus and (2) posterior cingulate or precuneus. Functional MRI data will be preprocessed with standard neuroimaging packages, including statistical parametric mapping 12, FSL, and the functional connectivity toolbox (CONN v17f or later; focusing on DMN network connectivity) following standard published pipelines [44].

#### Intervention: cTBS Versus Sham

Following the first MRI scan, participants exited the scanner and underwent either cTBS stimulation or sham stimulation according to their preassigned double-blinded condition. The stimulator was preprogrammed prior to the start of the session and the participants and technicians that administered the stimulation procedure were blind to the administered condition. Each individual was expected to have a different sensitivity to the magnetic fields generated by the stimulation coil, and the stimulation intensity was adjusted based on each individual’s resting motor threshold (RMT). The real-time, motor-evoked potential of muscle contraction is provided to ensure consistent force production. Once the RMT was identified, the stimulation intensity for TBS was set to 70% of each participant’s RMT.

**Figure 3:**
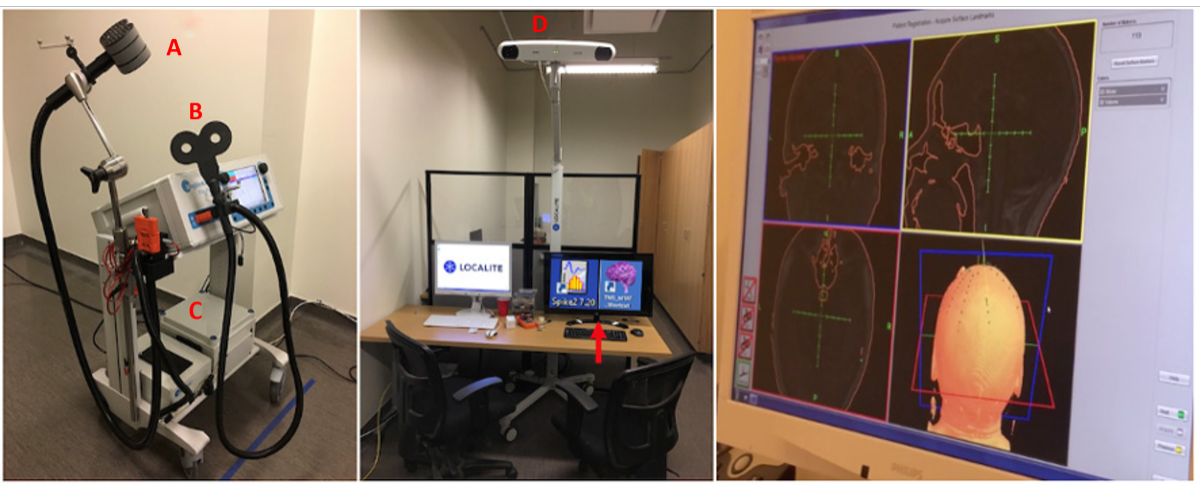
Transcranial magnetic intervention system set-up for the pilot randomized controlled trial. Left: (A) the continuous theta burst stimulation is administered with a MagVenture Cool-B65 stimulator that included an active and sham side; (B) the resting motor threshold was determined using a MagPro X100 stimulator with a figure-of-8 coil; (C) the transcranial magnetic stimulation system is maintained at a constant temperature using an active cooling system. Middle: the stimulation is directed by a computerized system that detected the orientation of the head in space using (D) an antenna camera and correlate it with the individual’s coregistered magnetic resonance imaging scan. Right: An image showing the Neuronavigation system used to administer the transcranial magnetic stimulation to the precise default mode network locations.

For primary rTMS stimulation, we located a predetermined node in the inferior parietal lobe, localized by coregistering the participant’s head, structural T1-weighted MRI image, and TMS coil in the same space using a TMS 3D Neuronavigation System. Once coregistration was complete, the Neuronavigation system provided real-time feedback on the TMS coil location and recorded the coil position and orientation relative to the head. The cTBS was applied using a figure-of-8 coil with an active cooling system connected to the MagPro magnetic stimulator (MagVenture Cool-B65). The same coil has 2 sides, 1 designed for active TMS stimulation, and the other is equipped with a magnetic shield that effectively blocks any stimulation and is used for the sham condition. The rTMS setup is shown in Figure 3.

For this project, we selected an easily accessible node of the DMN located on the left lateral parietal (LLP) cortex. The exact spatial location for stimulation is based on the Yeo et al [45] probability atlas. We downloaded this atlas into the Mango 4.1 visualization program (and identified the centroid of the LLP node of the DMN at the MNI coordinates of x (–48), y (–61.5), and z (32.5). These coordinates are converted into the same stereotaxic space as the participant’s brain to allow precision localization of the LLP node as the target for stimulation. Once this site was localized for the participants, a 40-second cTBS stimulation train (600 stimuli/session) or identically matched sham stimulation was administered using a MagVenture MagPro X100 stimulator (MagVenture Inc) connected with a figure-of-8 magnetic coil with active cooling.

#### Poststimulation Neuroimaging

Immediately following the cTBS or sham stimulation period, each participant underwent a second neuroimaging session that was identical to the prestimulation MRI session previously described.

#### PSG Recording

Participants slept undisturbed in a light- and temperature-controlled private bedroom while continuously monitored by a trained technician with a Nihon Kohden JE-921 PSG recording system using standard 10-20 electrode placement. Data from PSG were scored using Polysmith software according to the standard Rechtschaffen and Kales approach by a trained and certified sleep technician who was blind to the participant’s condition and status. The primary outcome metrics include sleep onset latency (SOL), latency to N1, N2, N3, and REM, total sleep time (TST), sleep efficiency (SE), wake after sleep onset (WASO), and number of awakenings and arousals, as well as time and percentage of time spent in each sleep stage.

#### Monitoring

Participants were assessed both prior to stimulation and after receiving stimulation for any adverse somatic, cognitive, or physical symptoms. Any presence of symptoms was logged in a HIPAA-compliant server and the participant was assessed by the study physician to determine if enrollment needed to be discontinued. All adverse events were reported to both IRB and OHRO. All study activity was subject to independent, random auditing from the Department of Psychiatry at the University of Arizona and study sponsors.

#### Data Management

All collected data were deidentified and stored within HIPAA-compliant, password-protected servers immediately after collection. Study data were only accessible to IRB-approved study staff. All data were checked and double-entered by study personnel no more than 1 week after data collection. Data quality checks were conducted every quarter by staff members for additional data assurance. Any data collected after participants’ consent were included in the study database for both compliant and noncompliant participants. However, following a per-protocol analysis, we are only using data from participants who fully completed the study due to within-subjects analysis.

### Primary Outcomes: FC Changes in the DMN

We hypothesize that active cTBS will disrupt the within-network and between-network connectivity of the DMN. This hypothesis will be assessed from a 10-minute resting-state FC scan and analyzed using standard procedures in the CONN (v17f or higher). Standard pipelines for preprocessing will be used. Within-network connectivity will be determined by first conducting an independent components analysis of the intrinsic activation patterns across the brain. Twenty independent components will be extracted. Mean activation clusters from each component will be examined for intercorrelation to determine within-network connectivity. Additionally, between-network, region-to-region, and seed-to-voxel analyses will be conducted to compare changes in FC between the 2 stimulation conditions.

### Analysis Plan

#### FC Changes to the DMN

First-level whole brain connectivity analyses will be undertaken in CONN for each subject at each of the 4 sessions (ie, active prestimulation, active poststimulation, sham prestimulation, and sham poststimulation). This will result in an FC map for each participant at each session, indicating the magnitude and direction of intrinsic correlation between each seed region (eg, lateral parietal cortex of the DMN) and all other voxels in the brain. For our primary analysis, we will estimate the effects of active cTBS on FC within the DMN using multimodal neuroimaging data including resting state FC. Raw NifTI images will be preprocessed using standard functional preprocessing pipelines in CONN [46].

The first-level connectivity maps will be combined in subsequent second-level analyses in CONN to explore the effects of cTBS stimulation on brain connectivity to include (1) independent components analysis, (2) seed-to-voxel analyses, and (3) region of interest to region of interest analyses [44]. Statistically significant differences in FC of the DMN between sham and active conditions will be the minimal important change to confirm the primary hypothesis. Next, PSG parameters measuring sleep stage duration and onsets, arousals, and awakenings will be added as covariates of FC change from before to after stimulation, comparing sham and active cTBS conditions.

#### PSG Analysis

We will assess the effects of active cTBS on sleep and whether participants demonstrated greater sleep quality during their active cTBS session compared with their sham stimulation session. Analyses will be conducted on sleep variables including total sleep time; N1 latency; N2 latency; N3 latency; REM latency; SOL; persistent SOL; SE; WASO; total number of awakenings; spontaneous arousals; wake duration; and stage duration for N1, N2, N3, and REM. Analyses will include repeated measures ANOVA controlling for covariates. Statistically significant difference between sham and active conditions will be the minimal important change to confirm this hypothesis.

#### Exploratory Analysis

We will examine the effects of active cTBS on secondary outcomes of cognitive performance, mood, and side effects compared with sham stimulation using repeated measures ANOVAs and nonparametric tests accordingly.

## Results

Our study was funded in September 2020 by the Department of Defense (W81XWH2010173). We completed the enrollment of our target population of 20 participants in October 2022. As of July 2023, we will initiate extensive neuroimaging data analysis and anticipate that the full results of the study will be published by 2024.

## Discussion

### Study Rationale

With a growing number of people reporting greater sleep disturbances, identifying effective nonpharmacological interventions is an important step in providing more innovative and noninvasive approaches for improving sleep outcomes. While the sample size for this study is small, this is the first study to investigate cTBS effects on the DMN to improve sleep outcomes.

### Experimental Clinical Design Rationale

Double-blind randomized controlled trials are the gold standard for scientific inquiry. Therefore, each participant will serve as their own control in a counterbalanced crossover design. Implementing a crossover design in which every participant receives both active and sham cTBS stimulation permits comparison of intraindividual differences in DMN connectivity and sleep quality parameters, as each participant acts as their own control. Because the goal was to examine the effects of cTBS on individuals with sleep problems, no healthy control samples were recruited or necessary to test our hypotheses. Blinding study personnel to the TMS condition also reduces systematic differences in the administration of the stimulation interventions and ostensibly reduces the influence of expectations about treatment effects.

### Brief cTBS of the LLP Lobule

Continuous theta burst was selected as the intervention based on its previously demonstrated inhibitory effects on the cortex [47]. A single 40-second cTBS session inhibits, or suppresses, neural excitability in the cortex for up to 50 minutes or longer [47]. Stimulation of a single node of the DMN is expected to inhibit local activity, with further suppression of cortical excitability propagating throughout the network overall. Inhibition of the left inferior parietal lobule was selected for two primary reasons: (1) prior research showed reduced metabolic activity in this region when individuals were falling asleep and (2) this region facilitates an easy-to-access node of the posterior DMN [48].

The localization of specific brain regions requires prestimulation imaging to account for individual variability in brain structure. For this reason, a critical element of that project was to show that stimulation of a single, easily accessible surface node of the larger DMN could lead to significant alterations in FC within this network. Prior investigations using cTBS have focused primarily on the stimulation of brain regions associated with the prefrontal areas of the DMN. Sleep-related parameters have rarely been collected as outcome measures for cTBS. One prior study examining rTMS effects on the right parietal cortex showed reduced anxiety and insomnia symptoms [49]. However, that study used a stimulation session (of three 1-Hz stimulations every 10 minutes) spread across 10 days. The presently proposed cTBS protocol is aimed at establishing initial research outcomes of the effects of a single burst of cTBS on disrupting network connectivity of the DMN to improve sleep. Thus, only a single stimulation session of both active cTBS and sham is needed to identify the intraindividual effects of cTBS on sleep outcomes.

### Limitations and Future Directions

Targeting a single node of the DMN and conducting only 1 stimulation session are limitations of this protocol to sufficiently answer the research questions and aims proposed regarding DMN and insomnia improvement. Future work could determine the most effective stimulation sites within the DMN and the optimal number of pulses and administrations to optimize this potential intervention for sleep problems. Larger samples are also needed than the small pilot sample collected for this protocol to examine the effects of sex observed in previous studies [38].

### Summary and Conclusions

Insomnia symptoms linked with many other psychiatric and physiological morbidities have become an increasingly prevalent and important health care and societal concern. Noninvasive neuromodulation can be inexpensive and is a technique accessible to many types of treatment providers. The protocol presented here unifies the prominent aspects of insomnia—physiological and psychological hyperarousal—by targeting a unique neural network, known to be associated with proposed mechanisms of insomnia [8,50].

## Abbreviations

ANCOVA: analysis of covariance
BDI-II: Beck Depression Inventory
CONN: Functional Connectivity Toolbox
cTBS: continuous theta burst stimulation
CVLT-3: California Verbal Learning Task 3
DMN: default mode network
FC: functional connectivity
GCTI: Glasgow Content of Thoughts Inventory
HIPAA: Health Insurance Portability and Accountability Act
IRB: Institutional Review Board
LLP: left lateral parietal
MRI: magnetic resonance imaging
OHRO: Office of Human Research Oversight
PSG: polysomnography
PVT: psychomotor vigilance task
RBANS: Repeatable Battery for the Assessment of Neuropsychological Status
RMT: resting motor threshold
rTMS: repetitive transcranial magnetic stimulation
SE: sleep efficiency
SOL: sleep onset latency
TMS: transcranial magnetic stimulation
TST: total sleep time
WASO: wake after sleep onset
